# Rethinking Risk for Pneumococcal Disease in Adults: The Role of Risk Stacking

**DOI:** 10.1093/ofid/ofv020

**Published:** 2015-03-05

**Authors:** Stephen I. Pelton, Kimberly M. Shea, Derek Weycker, Raymond A. Farkouh, David R. Strutton, John Edelsberg

**Affiliations:** 1 Boston University Schools of Medicine and Public Health; 2 Boston Medical Center; 3 Policy Analysis Inc., Brookline, Massachusetts; 4 Pfizer Inc., Collegeville, Pennsylvania

**Keywords:** comorbidity, pneumococcal infections, pneumonia, *Streptococcus* pneumonia

## Abstract

Using data from 3 private healthcare claims repositories, we evaluated the incidence of pneumococcal disease among adults with US Advisory Committee on Immunization Practices (ACIP) defined at-risk conditions or rheumatoid arthritis, lupus, Crohn's disease, and neuromuscular disorder/seizures and those with traditional high-risk conditions. We observed that adults with ≥2 concurrent comorbid conditions had pneumococcal disease incidence rates that were as high as or higher than rates observed in those with traditional high-risk conditions.

In a recent study, we detailed incidence rates and relative rates of pneumococcal disease among adults with chronic medical (“at-risk”) conditions and immunocompromising (“high-risk”) conditions in comparison with their healthy counterparts [1]. We identified that, among persons with at-risk conditions, rates of invasive pneumococcal disease (IPD), pneumococcal pneumonia, and all-cause pneumonia substantially increased with the accumulation of concurrent at-risk conditions, hereafter referred to as risk stacking. This brief report further clarifies both the magnitude of the increase in disease risk in adults with multiple at-risk conditions as well as the proportion of overall disease burden that these individuals contribute.

## METHODS

We used the same retrospective cohort design and 3 data sources—Truven Health Analytics MarketScan Commercial Claims and Encounters and Medicare Supplemental and Coordination of Benefits Databases, IMS LifeLink PharMetrics Health Plan Claims Database, and Optum Research Database—as described in our recent publication [1]. The study population comprised all adults aged ≥18 years who had medical and drug benefits from participating health plans on the first day of ≥1 calendar year from 2007 to 2010 and for at least 1 year before the beginning of the calendar year. Study subjects were stratified based on age (18–49, 50–64, and ≥65 years) and risk profile (at-risk, high-risk, and healthy). The at-risk category included immunocompetent persons with indications for adult pneumococcal vaccination [2], as identified by the US Advisory Committee on Immunization Practices, or other conditions hypothesized to increase risk for pneumococcal disease (rheumatoid arthritis, lupus, Crohn's disease, and neuromuscular disorder or seizures) in the absence of a high-risk condition. The high-risk group included immunocompromised and immunosuppressed persons (including those with chronic renal failure) and those with a cochlear implant. Episodes of nonbacteremic all-cause pneumonia, nonbacteremic pneumococcal pneumonia, and IPD from January 1st through December 31st of each study year were identified from diagnostic, procedure, and pharmacy claims. Rate ratios for disease episodes among persons with at-risk and high-risk conditions, respectively, compared with age-stratified healthy counterparts (ie, those without at-risk or high-risk conditions) were estimated using Poisson regression (SAS version 9.3). The precise methods, operational algorithms, and diagnosis, procedure, and drug codes that were used are detailed in previous publications from our group [1, 3].

## RESULTS

**Figure 1.**
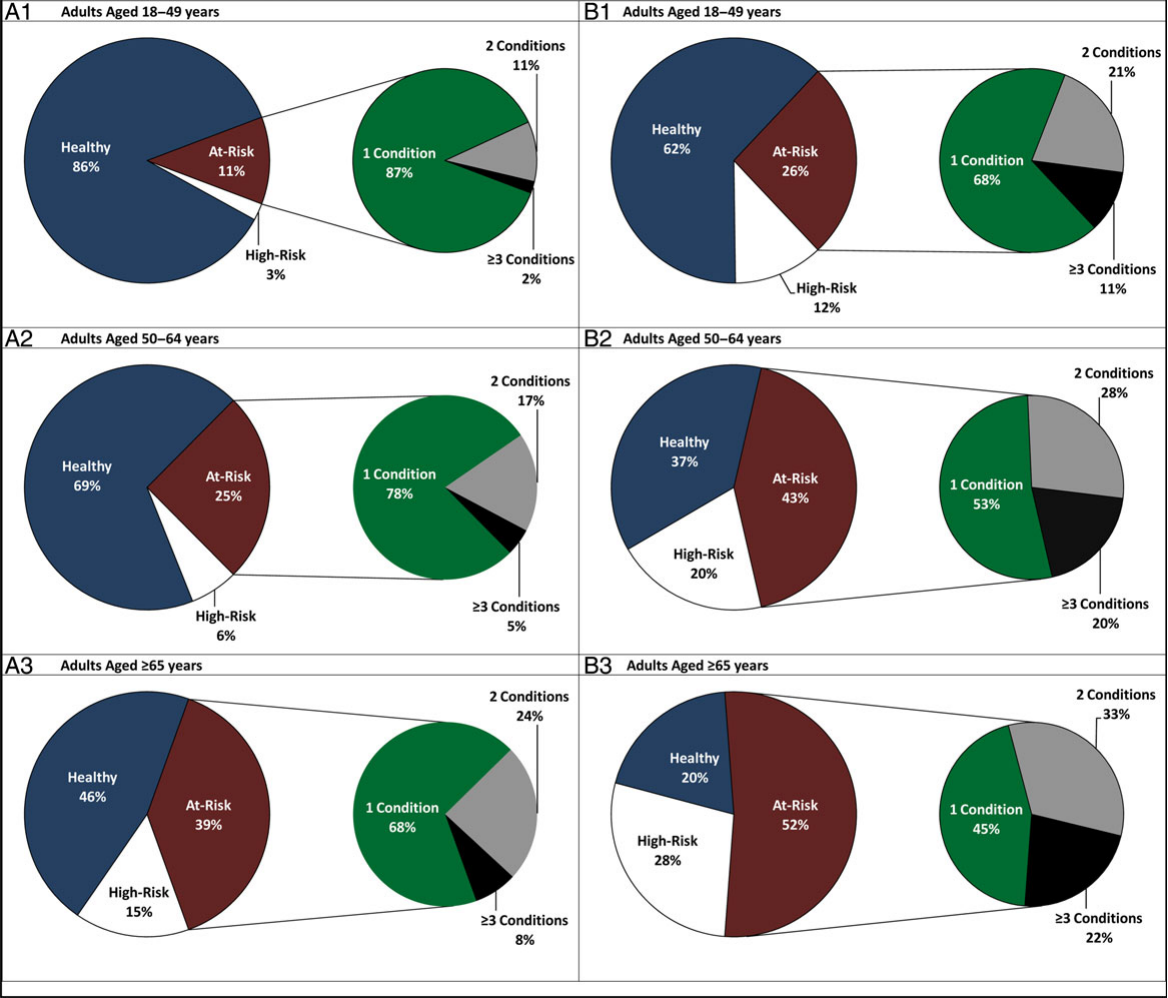
Distribution of adults by risk profile, and among at-risk adults, by number of conditions (ages 18–49 years [A1], ages 50–64 years [A2], ages ≥65 years [A3]). Distribution of pneumococcal pneumonia among adults by risk profile, and among at-risk adults, by number of conditions (ages 18–49 years [B1], ages 50–64 years [B2], ages ≥65 years [B3]).

We found that at least 1 at-risk condition, in the absence of a high-risk condition, was present in 19% of adults ≥18 years of age; a high-risk condition was present in 5% of the adult population. The prevalence of at-risk conditions (and high-risk conditions) increased with age: 11% of adults aged 18–49 years, 25% of adults aged 50–64 years, and 39% of adults aged ≥65 years had ≥1 at-risk condition (and no high-risk condition) (Figure 1A). Among adults with at-risk conditions, the percentage with ≥2 concurrent at-risk conditions also increased with age. Although diabetes and heart disease represented the most common combination of conditions among all adults, asthma in combination with diabetes was an important contributor in the 18- to 49-year-old age group, and chronic lung disease in combination with heart disease was an important contributor in those ≥65 years of age (Supplemental Table S1).

Figure 1B illustrates the proportion of pneumococcal pneumonia by risk profile in each age grouping. Individuals with high-risk conditions and those with ≥2 concurrent at-risk conditions each “suffered” a greater proportion of pneumococcal pneumonia episodes compared with their representation in the population. One and a half percent of adults 18–49 years of age had ≥2 concurrent at-risk conditions, yet they accounted for approximately 9% of all cases of pneumococcal pneumonia; among 50–64 year olds, 5.6% had ≥2 at-risk conditions and suffered 20% of all pneumococcal pneumonia; and for those ≥65 years of age, 12.4% of the population had ≥2 concurrent at-risk conditions but 29% of the pneumococcal pneumonia events.

**Figure 2.**
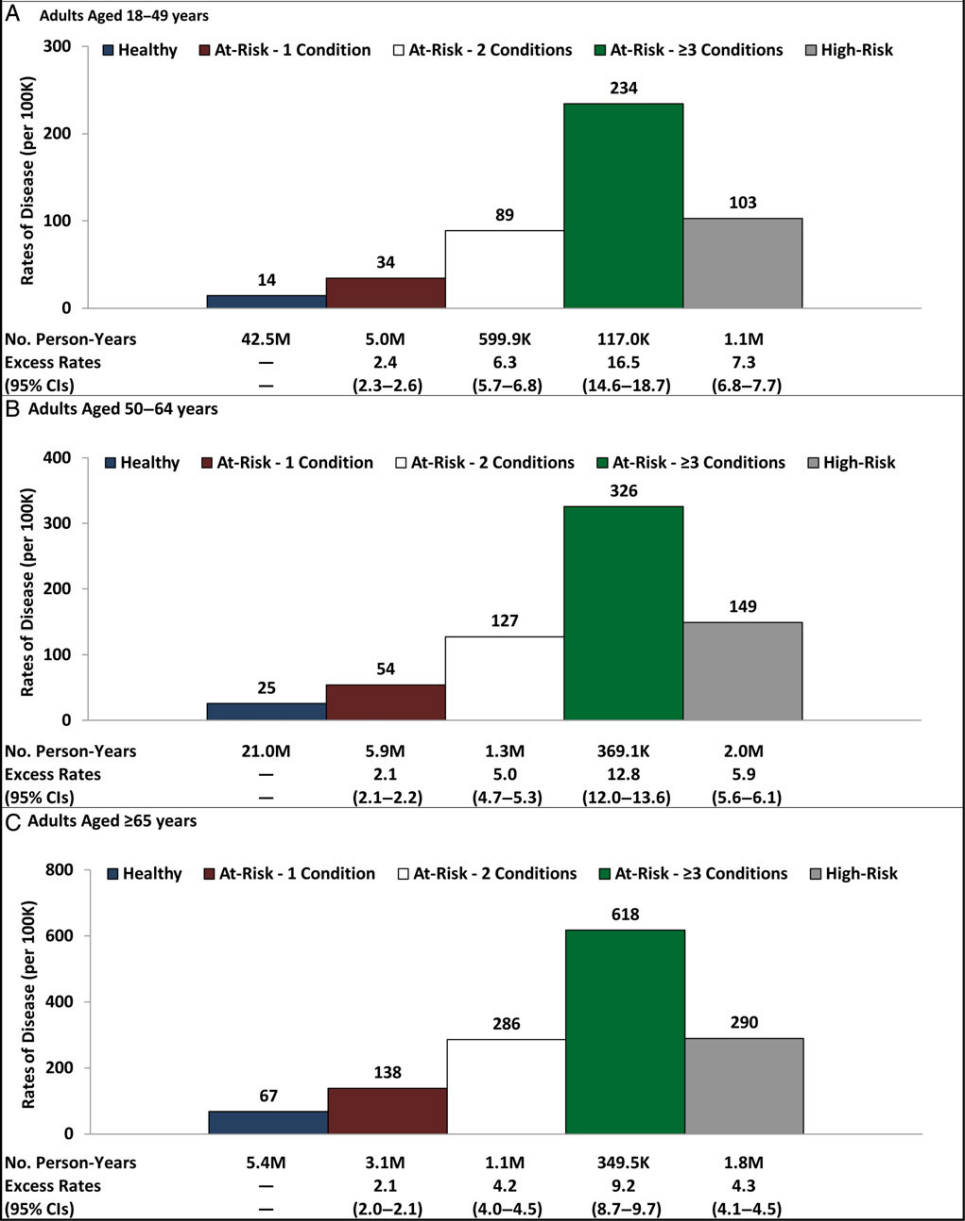
Rates of pneumococcal pneumonia (per 100K person-years) among adults by risk profile, and among at-risk adults, by number of conditions (ages 18–49 years [A], ages 50–64 years [B], ages ≥65 years [C]). Abbreviation: CI, confidence interval.

Figure 2 provides rates of pneumococcal pneumonia for otherwise healthy individuals, persons with ≥1 at-risk condition by the number of concurrent at-risk conditions, and those with high-risk conditions. The highest incidence of disease was observed in individuals with ≥3 at-risk conditions in each age group, exceeding that observed in high-risk individuals by approximately 2-fold. Although the incidence of IPD was lower, and the incidence of all-cause pneumonia was higher, than the incidence of pneumococcal pneumonia, distributions of disease burden and patterns of risk stacking were similar (data not shown).

## DISCUSSION

In adults ≥18 years of age, two thirds of pneumococcal disease is found in 25% of the population with high-risk and at-risk conditions. In particular, 22% of pneumococcal disease occurred in 5% of adults with high-risk conditions, and 43% occurred in 19% of adults with at-risk conditions. In each of the 3 age groups we studied, a disproportionate rate of pneumococcal disease was found in those with ≥2 concurrent at-risk conditions. We observed that adults with ≥2 comorbid conditions had pneumococcal disease incidence rates that were as high as, or higher than, rates observed in those with traditional high-risk conditions.

Because the combinations of comorbid conditions most often include conditions for which pneumococcal vaccination is already indicated, this study adds further support that the currently identified at-risk groups are appropriate targets for disease prevention [2]. However, because of the magnitude of increased risk identified in those persons with multiple comorbid conditions, if confirmed by other investigators, we suggest that such individuals be considered as high-risk. In addition, our findings have the following major implications if confirmed. The US population will continue to shift towards older age groups due to increasing life expectancy and falling reproductive rates [4]. Each of these demographic changes will result in an increasing proportion of persons with ≥2 comorbid conditions, and they will require new effective strategies for pneumococcal disease prevention. Individuals with comorbid conditions appear to be susceptible to a broad spectrum of pneumococcal serotypes as reflected by reports of increased disease (replacement) due to nonvaccine serotypes including those not currently in any vaccine formulations [5, 6, 7]. The question persists, however, as to why individuals with comorbid illness are at greater risk for pneumococcal disease? Epidemiologic and animal data suggest increased and disordered inflammation from aging and comorbidity leads to increased susceptibility to pneumococcal pneumonia, possibly related to an increased expression of bacterial ligands in the lung and delayed host responses as a result of chronic activation [8, 9]. The observed reduction in pneumonia fatalities in diabetics on statins has been hypothesized to result from the down regulation of immune activation due to their anti-inflammatory properties and/or a decrease in host cell lysis resulting from a reduction in circulating cholesterol-dependent cytotoxins such as pneumolysin [10–12].

Our study has several limitations, as reported in our prior publications and inherent in the use of healthcare claims data (errors of omission and commission in coding, and the absence of persons with no or public insurance). Second, the incidence of pneumococcal pneumonia (and IPD) in our population is lower than national estimates from the Centers for Disease Control and Prevention (CDC) [13]. However, our analysis of IPD incidence in our study population followed the same general age distribution as reported by the CDC, suggesting that the imperfect sensitivity of case ascertainment is proportional across age groups and would not impact rate ratios.

## CONCLUSIONS

In summary, our findings—based on 3 large and geographically diverse US populations—indicate that among adults with multiple at-risk conditions, pneumococcal disease rates are notably high and are comparable to those among adults with high-risk conditions. Moreover, although only a relatively small percentage of our study population had >1 concurrent at-risk condition, especially among the youngest age group, the percentage of US adults with multiple conditions is increasing, suggesting that risk stacking is likely to account for an increasing proportion of cases of pneumococcal disease.

## Supplementary Material

Supplementary material is available online at *Open Forum Infectious Diseases* (http://OpenForumInfectiousDiseases.oxfordjournals.org/).

Supplementary Data
