# Supplementary material for: Rethinking Risk for Pneumococcal Disease in Adults: The Role of Risk Stacking
Source: Open Forum Infect Dis. 2015 Mar 5;2(1):ofv020. doi: 10.1093/ofid/ofv020 (PMC4438900; doi:10.1093/ofid/ofv020)
Supplement: Supplementary Data [file supp_2_1_ofv020__index.html]

Rethinking Risk for Pneumococcal Disease in Adults: The Role of Risk Stacking — Rethinking Risk for Pneumococcal Disease in Adults: The Role of Risk Stacking — Supplementary Data 

# Rethinking Risk for Pneumococcal Disease in Adults: The Role of Risk Stacking

## Supplementary Data

Supplementary Data

**Files in this Data Supplement:**

- Supplementary Data - Docx file
