# Supplementary material for: Rethinking Risk for Pneumococcal Disease in Adults: The Role of Risk Stacking
Source: Open Forum Infect Dis. 2015 Mar 5;2(1):ofv020. doi: 10.1093/ofid/ofv020 (PMC4438900; doi:10.1093/ofid/ofv020)
Supplement: Supplementary file 2 [file supp_ofv020_ofv020supp.docx]

**Supplemental Table.** Distribution of all at-risk adults with ≥2 conditions, by combination of conditions (top 25 only)

| | Age (years) | | | |
|---|---|---|---|---|
| | >=18 | 18-49 | 50-64 | >=65 |
| Heart Disease + Diabetes | 30% | 12% | 29% | 39% |
| Heart Disease + Lung Disease | 8% | 3% | 5% | 13% |
| Heart Disease + Lung Disease + Diabetes | 5% | 1% | 3% | 8% |
| Asthma + Diabetes | 4% | 8% | 5% | 2% |
| Lung Disease + Asthma | 4% | 6% | 4% | 3% |
| Heart Disease + Smoker | 4% | 5% | 5% | 1% |
| Lung Disease + Diabetes | 4% | 3% | 4% | 5% |
| Lung Disease + Smoker | 4% | 5% | 5% | 2% |
| Diabetes + Smoker | 4% | 7% | 4% | 1% |
| Heart Disease + Asthma | 3% | 4% | 3% | 2% |
| Heart Disease + Lung Disease + Asthma | 2% | 1% | 1% | 3% |
| Asthma + Smoker | 2% | 6% | 1% | 0% |
| Diabetes + Liver Disease | 1% | 2% | 2% | 1% |
| Diabetes + Rheumatoid Arthritis, Lupus or Crohn's | 1% | 1% | 2% | 1% |
| Heart Disease + Lung Disease + Smoker | 1% | 1% | 2% | 1% |
| Heart Disease + Rheumatoid Arthritis, Lupus or Crohn's | 1% | 1% | 1% | 2% |
| Heart Disease + Asthma + Diabetes | 1% | 1% | 1% | 1% |
| Heart Disease + Lung Disease + Asthma + Diabetes | 1% | 0% | 1% | 2% |
| Heart Disease + Neuromuscular Disorder | 1% | 1% | 1% | 1% |
| Heart Disease + Diabetes + Smoker | 1% | 1% | 2% | 1% |
| Alcoholism + Smoker | 1% | 4% | 1% | 0% |
| Lung Disease + Asthma + Diabetes | 1% | 1% | 1% | 1% |
| Diabetes + Neuromuscular Disorder | 1% | 2% | 1% | 1% |
| Lung Disease + Asthma + Smoker | 1% | 1% | 1% | 0% |
| Lung Disease + Diabetes + Smoker | 1% | 0% | 1% | 0% |
